# Atorvastatin as a potential anti-malarial drug: in vitro synergy in combinational therapy with quinine against *Plasmodium falciparum*

**DOI:** 10.1186/1475-2875-9-139

**Published:** 2010-05-25

**Authors:** Véronique Parquet, Maud Henry, Nathalie Wurtz, Jerome Dormoi, Sébastien Briolant, Marine Gil, Eric Baret, Rémy Amalvict, Christophe Rogier, Bruno Pradines

**Affiliations:** 1 Unité de Recherche en Biologie et Epidémiologie Parasitaires - Unité de Recherche pour les Maladies Infectieuses et Tropicales Emergentes - UMR 6236, Institut de Médecine Tropicale du Service de Santé des Armées, Marseille, France; 2 Unité de Recherche en Physiologie et Pharmacocinétique Parasitaires - UMR-MD3 Relations Hôte-Parasites - Pharmacologie et Thérapeutique, Institut de Médecine Tropicale du Service de Santé des Armées, Marseille, France

## Abstract

**Background:**

Quinine (QN) remains the first line anti-malarial drug for the treatment of complicated malaria in Europe and Africa. The emergence of QN resistance has been documented. QN resistance is not yet a significant problem, but there is an urgent need to discover partners for use in combination with QN. The aim of the study was to assess the in vitro potentiating effects of atorvastatin (AVA), a 3-hydroxy-3-methylglutaryl coenzyme A (HMG-CoA) reductase inhibitor, in combination with QN against *Plasmodium falciparum *and to evaluate whether the effects of AVA could be associated with gene copy number or mutations in genes involved in QN resistance, such as *pfcrt*, *pfmdr1*, *pfmrp *and *pfnhe*.

**Methods:**

The susceptibilities to combination of AVA with QN were assessed against 21 parasite strains using the *in vitro *isotopic microtest. Genotypes and gene copy number were assessed for *pfcrt*, *pfmdr1*, *pfmdr2*, *pfmrp *genes. In addition, the number of DNNND, DDNHNDNHNN repeats in *pfnhe-1 *ms4760 and the ms4760 profile were determined for each strains of *P. falciparum*.

**Results:**

AVA demonstrated synergistic effects in combination with QN against 21 *P. falciparum *strains. The QN IC_50 _was reduced by 5% (0% to 15%; 95%CI: 1%-8%), 10% (3% to 23%; 95%CI: 7%-14%) and 22% (14% to 40%; 95%CI: 19%-25%) in presence of AVA at concentrations of 0.1, 0.5 and 1.0 μM, respectively. These reductions were all significant (p < 0.009). The reduction in the QN IC_50 _in presence of AVA was not significantly correlated with the QN IC_50 _(r = 0.22, *P *= 0.3288) or the AVA IC_50 _(r = 0.03, P = 0.8946). The synergistic effect of AVA in combination with QN was not significantly associated with polymorphisms in the *pfcrt*, *pfmdr1*, *pfmrp*, and *pfnhe-1 *genes that could be involved in QN resistance. The synergistic effect of AVA on QN responses was not significantly associated with *pfmdr1 *copy number (*P *= 0.0428).

**Conclusion:**

The synergistic effect of AVA in combination with QN was found to be unrelated to mutations occurring in transport protein genes involved in QN drug resistance. The different mechanisms of drug uptake and/or mode of action for AVA compared to the other anti-malarial drugs, as well as the AVA-mediated synergy of the anti-malarial effect of QN, suggests that AVA will be a good candidate for combinatorial malaria treatment. All of these observations support calls for both an in vivo evaluation with pharmacokinetic component and clinical trials of AVA as an anti-malarial therapy.

## Background

During the past 20 years, many strains of *Plasmodium falciparum *have become resistant to chloroquine and other anti-malarial drugs [1]. This has prompted a search for an effective alternative anti-malarial drug with minimal side effects. The emergence and spread of parasites resistant to anti-malarial drugs has resulted in an urgent need for discovery and development of novel anti-malarial compounds.

Quinine (QN) has been used as a malaria treatment for more than 350 years in Africa and remains the first-line anti-malarial drug for the treatment of complicated malaria in Europe and Africa. However, cases of QN clinical failure were observed in Brazil and Asia in the 1960s [2,3]. In the 1980s, clinical failures became more frequent in Southeast Asia, South America and Africa [4-8]. However, QN resistance is not yet a significant problem. QN continues to be widely used at present as a second-line therapy for uncomplicated malaria in Africa and other areas. Nevertheless, there is an urgent need for discovery partners for combination with QN.

Atorvastatin (AVA), a 3-hydroxy-3-methylglutaryl-coenzyme A (HMG-CoA) reductase inhibitor, belongs to a family of lipid-lowering drugs that are currently used for the control of hyperlipidaemia and are considered useful for protection from cardiovascular events. Apart from the cholesterol-lowering activity of statins, the immunomodulatory and pleiotropic effects of statins may significantly impact infection-related survival [9,10]. AVA reduced the intracellular growth of *Salmonella typhimurium *in macrophages in a mouse model [11]. Additionally, AVA has an anti-hepatitis C virus (HCV) effect and enhanced the anti-HCV effect of interferon when used in combination [12]. However, the use of statin alone in patients with, or at risk of, severe infections is contentious [13]. Preoperative statin use was not associated with a reduction of the rate of postoperative infection among patients who underwent cardiac surgery.

Statins severely interfered with the growth of protozoan parasites in the Trypanosomatidae family, such as *Trypanosoma cruzi *and various *Leishmania *species [14-16]. Additionally, an HMG-CoA reductase has been detected in *Trypanosoma *and *Leishmania *[16].

AVA demonstrated anti-malarial activity in vitro even though an HMG-CoA homolog was not identified by BLASTX analysis of the *P. falciparum *sequence with other protozoal HMG-CoA protein sequences [17]. AVA is ten-fold more active against *P. falciparum *strains than other statins [17]. Additionally, AVA demonstrated no in vitro cross-resistance with quinine, chloroquine, monodesethylamodiaquine, mefloquine, lumefantrine, dihydroartemisinin, atovaquone, or doxycycline, and the IC_50 _values for AVA were found to be unrelated to mutations in transport protein genes involved in quinoline anti-malarial drug resistance, such as *pfcrt*, *pfmdr1*, *pfmrp*, and *pfnhe-1 *[18]. AVA had synergistic effects in combination with dihydroartemisinin [19].

AVA is also an inhibitor of phosphoglycoprotein (Pgp), an efflux protein in cancer cells [20-22]. Multi-drug resistance (MDR)-like proteins are involved in quinoline resistance in *P. falciparum *[23-25]. PfMRP is involved in QN resistance [26,27].

The objectives of this study were 1) to assess the in vitro potentiation effects of AVA in combination with QN against 21 strains of *P. falciparum *that were isolated from a wide panel of countries and had different drug susceptibility profiles, and 2) to evaluate if AVA could be an inhibitor of *P. falciparum *MDR-like proteins, such as Pgh1, PfMRP or PfMDR2, or transporters involved in drug resistance, such as PfCRT (*P. falciparum *chloroquine resistance transporter) [28] and PfNHE-1 (*P. falciparum *sodium/hydrogen exchanger) [29,30].

## Methods

### *Plasmodium falciparum *cultures

Twenty-one parasite strains or clones from a wide panel of countries (Brazil, Cambodia, Cameroon, Djibouti, French Guyana, the Gambia, Honduras, Indochina, Niger, Republic of Comoros, Republic of the Congo, Senegal, Sierra Leone, Sudan, and Uganda) were maintained in culture in RPMI 1640 (Invitrogen, Paisley, UK) supplemented with 10% human serum (Abcys S.A. Paris, France) and buffered with 25 mM HEPES and 25 mM NaHCO_3_. Parasites were grown in type A^+ ^human red blood cells under controlled atmospheric conditions (10% O_2_, 5% CO_2, _and 85% N_2_) at 37°C with a humidity of 95%. All strains were synchronized twice with sorbitol before use [31]. Clonality was verified using PCR genotyping of polymorphic genetic markers, *msp1*, *msp2*, and microsatellite loci [32,33]. The potentiation evaluation for each strain was assessed in three independent experiments.

### Drugs

AVA calcium salt was purchased from Molekula (UK). QN was purchased from Sigma (Saint Louis, MO). AVA was dissolved in 1% DMSO (v/v) in RPMI. Two-fold serial dilutions, with final concentrations ranging from 0.01 μM to 200 μM, were prepared in 1% DMSO in RPMI and plated in 96-well plates just before use. QN was dissolved first in methanol and then diluted in water to obtain final concentrations ranging from 5 to 3400 nM.

### *In vitro *assay

In order to assess the synergy of AVA with QN, 25 μl of AVA per well and 200 μl of the suspension of synchronous parasitized red blood cells (final parasitaemia, 0.5%; final haematocrit, 1.5%) per well were plated in 96-well plates containing the QN concentrations. The in vitro isotopic micro-test used had been previously described [34].

The drug concentration able to inhibit 50% of parasite growth (IC_50_) was calculated as the drug concentration corresponding to 50% of the incorporation of tritiated hypoxanthine by the parasite in the drug-free control wells. The IC_50 _value was determined using the non-linear regression analysis of log-based dose-response curves (Riasmart, Packard, Meriden, USA).

In order to evaluate the modulation of QN resistance by AVA, isobolograms were constructed by plotting a pair of fractional IC_50 _for each combination of AVA and QN for both parasite strains. The AVA fractional IC_50 _was calculated by dividing their fixed concentrations by the IC_50 _of tested drugs alone and plotted on the horizontal axis. The QN fractional IC_50 _was calculated by dividing the IC_50 _of QN combined with fixed concentrations of AVA and plotted on the vertical axis. Points lying above the diagonal line (corresponding to the points where there is no interaction between the drugs) are antagonistic, points below the diagonal line are considered to be synergistic.

The interaction parameter indicating relative degree of interaction (I) was calculated using the equation Y_i _= 1-[X_i_/(X_i _+ e^i^_* _(1 - X_i_))], where Y_i _= IC_50 _for AVA when combined with QN and X_i _= IC_50 _for QN when combined with AVA [35]. This representation of drug interaction provides a single quantitative estimate of relative degree of interaction even when the experimental ratios are not optimum. Positive values of I indicate synergism, negative values antagonism; addition occurs when it equals zero.

In addition, three concentrations of AVA (0.1, 0.5 and 1 μM), which were relevant with AVA plasma concentrations achievable in patients taking 80 mg of AVA daily [36], were reanalysed separately. The concentration of AVA that reduced the IC_50 _of QN by 50% when used alone, [AVA]_QN_, was calculated for each strain of *P. falciparum*.

### Nucleic acid extraction

The total genomic DNA of each strain was isolated using the E.Z.N.A. Blood DNA kit (Omega Bio-Tek, GA, U.S.A.). The RNA of each strain was purified using the QIAamp Blood Mini kit (QIAGEN, Germany). The methods for the SNPs identification of *pfcrt *[37], *pfmdr1 *[38], *pfmdr2 *[38] and *pfmrp *[38], for the identification of *pfnhe-1 *microsatellite profiles [29,37] and for the estimation of the copy number of *pfmdr1 *and *pfmdr2 *[38] were previously described.

### Statistical analysis

The differences in MQ IC_50 _between AVA concentrations groups have first been tested using ANOVA for repeated measures to take into account the fact that observations made within each strain were not independent. Using the most conservative correction for interdependence between observations (*i.e*. Box's conservative epsilon), the differences in QN IC_50 _were tested for concentrations relevant with AVA plasma concentrations achievable in patients taking 80 mg of AVA daily (0.1, 0.5 and 1 μM). Using a random effect linear regression approach, the regression coefficients for the log-transformed QN IC50% indicated the significance of the mean fold change in QN IC_50 _when adding AVA concentrations of 0.1, 0.5 and 1.0 μM.

The Kruskal-Wallis test or the Mann-Whitney U test of was used, when appropriate, to compare the equality of the populations for each mutation. The results of these tests were compared according to the alleles at each locus. The differences in IC_50 _for QN were then tested 22 times (*i.e*., one per locus). The probability of getting a significant result with 22 tests at the α = 0.05 level of significance was 1-0.95^22 ^(1-probability of not getting a significant result with 22 tests). According to the Bonferroni correction, it was concluded that a difference was significant when at least one of the 22 comparisons yielded a significance level below 0.05/22 = 0.0023.

## Results

### Results of in vitro potentiation

**Figure 1:**
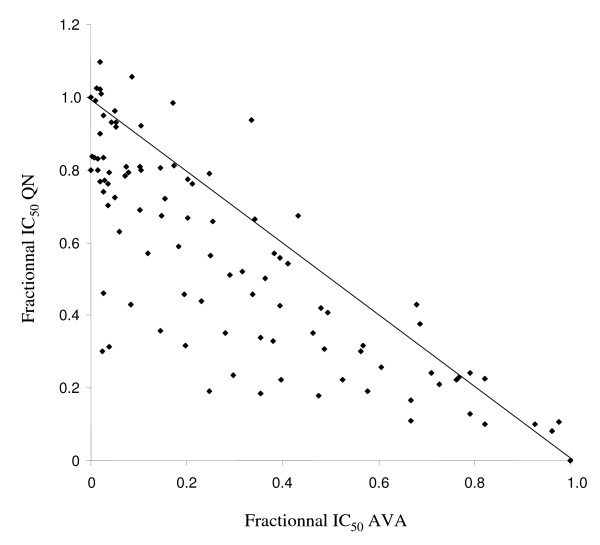
**In vitro synergistic interactions between atorvastatin (AVA) and quinine (QN) against the 21 strains of *P. falciparum***.

AVA demonstrated synergistic effects in combination with QN (Figure 1). The differences in QN IC_50 _between AVA concentrations groups have first been tested using ANOVA for repeated measures to take into account the fact that observations made within each strain were not independent. Using the most conservative correction for interdependence between observations (*i.e*. Box's conservative epsilon), the differences in QN IC_50 _were highly significant (p < 0.009) at concentrations relevant with AVA plasma concentrations achievable in patients taking 80 mg of AVA daily (Table 1). Using a random effect linear regression approach, the regression coefficients for the log-transformed QN IC50% indicated that the mean fold change in QN IC_50 _when adding AVA concentrations of 0.1, 0.5 and 1.0 μM (0.95; 0.90 and 0.78, respectively) were also highly significant (p < 0.009). The QN IC_50 _was reduced by 5% (0% to 15%; 95%CI: 1%-8%), 10% (3% to 23%; 95%CI: 7%-14%) and 22% (14% to 40%; 95%CI: 19%-25%) in presence of AVA at concentrations of 0.1, 0.5 and 1.0 μM, respectively. These reductions were all significant (p < 0.009). Another finding was that the synergy of the effects of QN was AVA dose-dependent. The reductions in QN IC_50 _were also significant between 0.1 AVA and 0.5 AVA (p < 0.0014) and between 0.5 AVA and 1.0 AVA (p < 0.0001).

**Table 1:** In vitro susceptibility of *Plasmodium falciparum *strains to atorvastatin alone (AVA), quinine alone (QN) and the combination of QN + AVA at concentrations of 0.1 μM, 0.5 μM and 1.0 μM.

Strains	IC_50 _AVA alone	IC_50 _QN alone	IC_50 _QN + AVA 0.1 μM	IC_50 _QN + AVA 0.5 μM	IC_50 _QN + AVA 1.0 μM
IC_50 _geometric mean	7.24 μM	359 nM	341 nM	321 nM	280 nM
IC_50 _mean 95% CI	6.68-7.86	216-596	203-574	190-542	165-474
Average IC_50 _diminution			5%	10%	22%
Average IC_50 _diminution 95%CI			1%-8%	7%-14%	19%-25%
P-value (*vs *QN alone)			0.009	< 0.001	< 0.001

95%CI: 95% Confident Interval

Eleven strains (53%) had interaction parameter indicating relative degree of interaction (I) > 1 (synergism) and 7 (33%) with 0 < I < 1 (weak synergism). Three strains (14%) had I ranging from -1 to 0 (weak antagonism).

The concentrations of AVA that reduced the IC_50 _of QN when used alone by 50% ([AVA]_QN_) ranged from 1.1 to 13.0 μM (geometric mean = 3.7 μM and 95% confidence interval = 2.8-5.1). The reduction of the QN IC_50 _in presence of AVA was not significantly correlated with the QN IC_50 _(r = 0.22, *P *= 0.3288) or the AVA IC_50 _(r = 0.03, P = 0.8946).

### Genotypes and gene copy number of the 21 strains of *P. falciparum*

The following mutations were identified in at least one strain: *pfcrt *M74I, N75E, K76T, A220S, Q271(E/V), N326S, I356T, and I371R; *pfmrp *H191Y and S437A; *pfmdr1 *N86Y, Y184F, S1034C, N1042D, and D1246Y; and pfmdr2 S208N and F423Y. Eight different ms4760 microsatellite profiles of *pfnhe-1 *were observed. The number of DNNND and DDNHNDNHNN repeats in ms4760 ranged from one to four and one to two, respectively. The copy number of *pfmdr1 *ranged from one to three. Only one copy of *pfmdr2 *was found in all of the 21 *P. falciparum *strains.

### Association of in vitro responses (IC_50_) and polymorphisms of genes

The association between polymorphism in *pfcrt *gene (codons 75, 76 and 220, *P *< 0.0022) or in *pfmrp *gene (codons 191 and 437, P = 0.0008) and QN responses, used alone, was highly significant (Additional file 1). The associations between ms4760 profiles, number of DNNND repeats or polymorphism in *pfmdr1 *gene (codons 1034 and 1042) and QN responses were not significant (*P *< 0.05 but > 0.0023) (Additional file 1). The synergistic effect of AVA on QN responses was not significantly associated with polymorphisms in the *pfcrt*, *pfmdr1*, *pfmrp *and *pfnhe-1 *genes (Additional file 1).

### Association of in vitro responses (IC_50_) and gene copy number

The association between *pfmdr1 *copy number and QN responses was not significant (*P *= 0.0086) (Additional file 1). The synergistic effect of AVA on QN responses was not significantly associated with *pfmdr1 *copy number (*P *= 0.0428) (Additional file 1).

## Discussion

Atorvastatin (AVA), a 3-hydroxy-3-methylglutaryl coenzyme A (HMG-CoA) reductase inhibitor, inhibited the in vitro growth of *P. falciparum *at the micromolar range. Whereas the generally agreed upon level of efficacy is in the low to mid-nanomolar range, if the mechanism of action of a novel anti-malarial compound was sufficiently new and different than those of the commonly used anti-malarial drugs, this compound would warrant further study. Nevertheless, AVA, used alone at 20 mg/kg of body weight, failed to prevent death from cerebral malaria or to effect parasitaemia in infected mice [39,40].

A large amount of scientific effort is spent on elucidating the mechanisms underlying the resistance to anti-malarial drugs with the hope of restoring/improving the efficacy of existing drugs and developing new drugs that can bypass the resistance mechanisms. One of the strategies that has been used to reduce the prevalence of malaria is the use of drug combinations. The combination protects each drug from the development of resistance and reduced the overall transmission rate of malaria [41]. The absence of in vitro cross-resistance between AVA and QN or the other anti-malarial drugs suggested that AVA could be a good potential partner for these anti-malarial drugs [18]. These different considerations and the capacity of AVA to inhibit human Pgh [22] lead us to evaluate AVA in combination with QN. AVA potentiated the activity of QN against 86% of the *P. falciparum *parasites tested (53% of synergism and 33% of weak synergism). However, AVA did not improve the QN responses of the totality of the strains tested. The potentiation was independent of the QN susceptibility level. AVA improved the in vitro activity of QN at concentrations relevant with AVA plasma concentrations achievable in patients taking 80 mg of AVA daily [36]. Furthermore, the administration doses of AVA in humans could be increased to 120 mg daily with only limited additional side effects [42]. A dose of 120 mg of AVA increased the maximal plasma concentration of Cmax after oral administration by levels from 4 to 10 fold. Cerebral malaria shares common pathophysiological features with sepsis, especially with regard to the pathology of the endothelium [43], and critically ill patients with sepsis had a significantly higher Cmax when compared to the healthy volunteers (110.5 vs. 5.9 ng/ml) [44]. The most important adverse events related to the use of atorvastatin are muscle toxicity and the effects on liver enzymes. However, rhabdomyolysis and creatinine kinase or transaminase level were not significantly increased in trials of intensive statin therapy or at high dose of AVA (80 mg/day) for periods ranging from 2 weeks to 5 years and were not age related [45-48]. The administration doses of AVA in humans at 120 mg daily did not induce rhabdomyolysis [42]. Cases of rhabdomyolysis were observed when AVA was co-administrated with other drugs, such as fusidic acid or ezetimibe or in patients with nephrotic syndrome. Biochemical evidence of skeletal muscle damage was common in malaria, but rhabdomyolysis appeared to be rare [49-51]. Rhabdomyolysis should be carefully survey in co-administration of AVA in malaria.

Nevertheless, these effects were not associated with polymorphisms in the genes involved in QN resistance such as *pfcrt *[28,52], *pfmdr1 *[53], *pfmrp *[26,54], or *pfnhe-1 *[29,30,55]. The synergistic effect of AVA in combination with QN was found to be unrelated to mutations occurring in the transport protein genes involved in quinoline drug resistance. In addition, the synergistic effect of AVA on QN responses was not significantly associated with *pfmdr1 *copy number but, as QN resistance, the significance level of this association was above the Bonferroni-corrected *P *value threshold (0.0023) but below 0.05. These data suggested that AVA could interfere with Pgh1, as predicted by the inhibition of Pgh in human cells by AVA [20-22]. However, this is a preliminary report. Twenty-one strains may not be sufficient to make definite conclusions.

Synergy of QN with AVA is a promising result in malaria treatment. QN remains the first line anti-malarial drug for the treatment of complicated malaria in Europe and Africa. However, clinical failures became more frequent in Southeast Asia, South America and Africa [4,6,8]. Artemisinin-based combination therapy (ACT) was proposed as a first-line treatment for uncomplicated malaria six years ago. However, individual *P. falciparum *isolates resistant to artemisinin *in vitro *and the first clinical failures have been described in Cambodia [56-60]. This emergence of parasite resistance to ACT indicates that novel compounds and combinations need to be discovered and developed. These data suggest that AVA will be a good candidate for combination with QN in malaria treatment.

## Conclusions

The mechanism underlying the anti-malarial activity of AVA is currently unknown. The absence of cross-resistance with QN, chloroquine, monodesethylamodiaquine, mefloquine, lumefantrine, dihydroartemisinin, atovaquone, or doxycycline suggests a different mechanism of drug uptake and/or mode of action for AVA [18]. AVA is a 3-hydroxy-3-methylglutaryl coenzyme A reductase inhibitor. Nevertheless, an HMG-CoA homolog was not identified by BLASTX analysis of the *P. falciparum *genome with other protozoal HMG-CoA proteins sequences. Parasites treated with mevastatin demonstrated depressed biosynthesis of dolichol and isoprenoid pyrophosphate [61]. In addition, mevastatin decreased the viability of cells through inhibition of the proteasome. One of the future objectives is to identify modifications of the *P. falciparum *proteome in parasites treated with AVA.

The different mechanisms of drug uptake and/or mode of action for AVA compared to the other anti-malarial drugs, as well as the AVA-mediated potentiation of the anti-malarial effect of QN, suggests that AVA will be a good candidate for combinatorial malaria treatment. All of these observations support calls for both an in vivo evaluation with pharmacokinetic component and clinical trials of AVA as an anti-malarial therapy.

## Conflict of interests

The authors have no conflicts of interest concerning the work reported in this paper. The authors do not own stocks or shares in a company that might be financially affected by the conclusions of this article.

## Authors' contributions

VP, MH, NW, SB and BP conceived and designed the experiments. VP, JD, EB and RA performed the in vitro experiments. MH, NW, SB and MG performed the genotyping. SB, CR and BP analysed the data. NW, SB, CR and BP wrote the paper. All authors read and approved the final manuscript.

## Supplementary Material

Additional file 1 Association of atorvastatin (AVA) in vitro responses (IC_50_), quinine (QN), AVA + QN, and polymorphisms in the *pfnhe-1*, *pfcrt*, *pfmdr1*, *pfmdr2 *and *pfmrp *genes or copy number of the *pfmdr2 *gene of 21 strains of *Plasmodium falciparum*. Click here for file

## Ackowledgments

This work was supported by the Direction Centrale du Service de Santé des Armées (grant no 2007 RC 32).
