# Supplementary material for: Atorvastatin as a potential anti-malarial drug: in vitro synergy in combinational therapy with quinine against Plasmodium falciparum
Source: Malar J. 2010 May 25;9:139. doi: 10.1186/1475-2875-9-139 (PMC2882376; doi:10.1186/1475-2875-9-139)
Supplement: Additional file 1 — Association of atorvastatin (AVA) in vitro responses (IC50), quinine (QN), AVA + QN, and polymorphisms in the pfnhe-1, pfcrt, pfmdr1, pfmdr2 and pfmrp genes or copy number of the pfmdr2 gene of 21 strains of Plasmodium falciparum. [file 1475-2875-9-139-S1.DOC]

**Table AF1: Association of atorvastatin (AVA) in vitro responses (IC_50_), quinine (QN), AVA + QN, and polymorphisms in the *pfnhe-1*, *pfcrt*, *pfmdr1*, *pfmdr2* and *pfmrp* genes or copy number of the *pfmdr2* gene of 21 strains of *Plasmodium falciparum***

| Genotype | AVA | | QN | | AVA + QN | |
| --- | --- | --- | --- | --- | --- | --- |
|  | P-value | Significance* | P-value | Significance* | P-value | Significance* |
| *pfnhe-1* ms4760 profiles | 0.4365 | NS | 0.0095 | NS | 0.5686 | NS |
| *pfnhe-1*, number of DNNND repeats | 0.1815 | NS | 0.0189 | NS | 0.4105 | NS |
| *pfnhe-1*, number of DDNHNDNHNN repeats | 0.0874 | NS | 0.0804 | NS | 0.1913 | NS |
| Mutation in codon 72 of *pfcrt* gene | 0.862 | NS | 0.8688 | NS | 0.7411 | NS |
| Mutation in codon 74 of *pfcrt* gene | 0.7159 | NS | 0.0024 | NS | 0.4913 | NS |
| Mutation in codon 75 of *pfcrt* gene | 0.7159 | NS | 0.0015 | S | 0.4913 | NS |
| Mutation in codon 76 of *pfcrt* gene | 0.5604 | NS | 0.0005 | S | 0.2615 | NS |
| Mutation in codon 220 of *pfcrt* gene | 0.7643 | NS | 0.0022 | S | 0.5757 | NS |
| Mutation in codon 271 of *pfcrt* gene | 0.5062 | NS | 0.0189 | NS | 0.5790 | NS |
| Mutation in codon 326 of *pfcrt* gene | 0.9458 | NS | 0.0058 | NS | 0.1526 | NS |
| Mutation in codon 356 of *pfcrt* gene | 0.8957 | NS | 0.0828 | NS | 0.8683 | NS |
| Mutation in codon 371 of *pfcrt* gene | 0.7159 | NS | 0.0024 | NS | 0.4913 | NS |
| Mutation in codon 86 of *pfmdr1* gene | 0.4792 | NS | 0.2313 | NS | 0.7513 | NS |
| Mutation in codon 184 of *pfmdr1* gene | 0.5613 | NS | 0.6203 | NS | 0.5796 | NS |
| Mutation in codon 1034 of *pfmdr1* gene | 0.6136 | NS | 0.0120 | NS | 0.6152 | NS |
| Mutation in codon 1042 of *pfmdr1* gene | 0.5892 | NS | 0.0316 | NS | 0.7881 | NS |
| Mutation in codon 1246 of *pfmdr1* gene | 0.3125 | NS | 0.3657 | NS | 0.4212 | NS |
| *Pfmdr1* copy number (1, 2 and 3) | 0.4096 | NS | 0.0315 | NS | 0.1199 | NS |
| *Pfmdr1* copy number (1 and > 1) | 0.2387 | NS | 0.0086 | NS | 0.0428 | NS |
| Mutation in codon 208 of *pfmdr2* gene | 0.9621 | NS | 0.0726 | NS | 0.7408 | NS |
| Mutation in codon 423 of *pfmdr2* gene | 0.5892 | NS | 0.2099 | NS | 0.2441 | NS |
| Mutation in codon 191 of *pfmrp* gene | 0.4751 | NS | 0.0008 | S | 0.0816 | NS |
| Mutation in codon 437 of *pfmrp* gene | 0.4751 | NS | 0.0008 | S | 0.0816 | NS |

U Test of Mann-Whitney or Kruskal-Wallis

significance cut-off = 0.0023 (0.05/22, 22 tests, correction of Bonferroni)

S = significant NS = non significant
